# Human umbilical cord mesenchymal stem cell-derived exosomes promote murine skin wound healing by neutrophil and macrophage modulations revealed by single-cell RNA sequencing

**DOI:** 10.3389/fimmu.2023.1142088

**Published:** 2023-03-06

**Authors:** Yuanyuan Liu, Mingwang Zhang, Yong Liao, Hongbo Chen, Dandan Su, Yuandong Tao, Jiangbo Li, Kai Luo, Lihua Wu, Xingyue Zhang, Rongya Yang

**Affiliations:** ^1^ Medical School of Chinese People’s Liberation Army, Beijing, China; ^2^ Department of Dermatology, the Seventh Medical Center of Chinese People's Liberation Army (PLA) General Hospital, Beijing, China; ^3^ Department of Dermatology, Southwest Hospital, Army Medical University, Chongqing, China; ^4^ School of Pharmaceutical Sciences, Sun Yat-sen University, Shenzhen, China; ^5^ Department of Pediatric Urology, the Seventh Medical Center of Chinese People's Liberation Army (PLA) General Hospital, Beijing, China; ^6^ Bioinformatics Center of Academy of Military Medical Sciences, Beijing, China; ^7^ Biomedical Treatment Center, the Seventh Medical Center of Chinese People's Liberation Army (PLA) General Hospital, Beijing, China

**Keywords:** exosomes, wound healing, single-cell RNA sequencing, cellular heterogeneity, neutrophils, macrophages

## Abstract

**Introduction:**

Full-thickness skin wound healing remains a serious undertaking for patients. While stem cell-derived exosomes have been proposed as a potential therapeutic approach, the underlying mechanism of action has yet to be fully elucidated. The current study aimed to investigate the impact of exosomes derived from human umbilical cord mesenchymal stem cells (hucMSC-Exosomes) on the single-cell transcriptome of neutrophils and macrophages in the context of wound healing.

**Methods:**

Utilizing single-cell RNA sequencing, the transcriptomic diversity of neutrophils and macrophages was analyzed in order to predict the cellular fate of these immune cells under the influence of hucMSC-Exosomes and to identify alterations of ligand-receptor interactions that may influence the wound microenvironment. The validity of the findings obtained from this analysis was subsequently corroborated by immunofluorescence, ELISA, and qRT-PCR. Neutrophil origins were characterized based on RNA velocity profiles.

**Results:**

The expression of *RETNLG* and *SLC2A3* was associated with migrating neutrophils, while *BCL2A1B* was linked to proliferating neutrophils. The hucMSC-Exosomes group exhibited significantly higher levels of M1 macrophages (215 vs 76, p < 0.00001), M2 macrophages (1231 vs 670, p < 0.00001), and neutrophils (930 vs 157, p < 0.00001) when compared to control group. Additionally, it was observed that hucMSC-Exosomes elicit alterations in the differentiation trajectories of macrophages towards more anti-inflammatory phenotypes, concomitant with changes in ligand-receptor interactions, thereby facilitating healing.

**Discussion:**

This study has revealed the transcriptomic heterogeneity of neutrophils and macrophages in the context of skin wound repair following hucMSC-Exosomes interventions, providing a deeper understanding of cellular responses to hucMSC-Exosomes, a rising target of wound healing intervention.

## 1 Introduction

Effectively accelerating cutaneous wound healing remains a pressing challenge. Cutaneous wound repair consists of a series of closely linked and overlapping processes involving multiple cells that synergistically regulate inflammation, proliferation, and remodeling. Acute full-thickness skin injury immediately triggers a rapid immune system response, with neutrophils first recruited to the wound in response to signals such as chemokines, calcium, and hydrogen peroxide, serving as the first line of defense against pathogenic microorganisms by releasing proteases, generating neutrophil extracellular traps, and phagocytosis (1). In the late inflammatory phase, neutrophils are phagocytosed by macrophages mainly through endocytosis. After 2-3 days of injury, monocytes are recruited and transformed into macrophages at the wound site, which fights infection mainly by releasing pro-inflammatory cytokines (IL-6, TNF-α, IL-1B) as well as by phagocytosis of pathogens (2). Synergistic regulation of neutrophils and immune cells is essential for wound repair. The relationship between the diversity of neutrophils and macrophages within wounds and their differentiation pathways and functions remain unclear.

In recent years, mesenchymal stem cells (MSCs) have been widely shown to be effective in promoting wound healing, however, stem cell treatments have a high carcinogenic risk and are prone to complications such as vascular embolism (3, 4). Exosomes, the main effector of MSCs paracrine production, are thought to have more stable biological effects, higher transduction efficiency, and easier quantification and storage (5). Studies have shown that human umbilical cord mesenchymal stem cells derived exosomes (hucMSC-Exosomes) can promote wound regeneration and repair by improving immune regulation, stimulating angiogenesis, promoting keratinocyte proliferation, and mediating extracellular matrix remodeling (6, 7). Although evidence exists regarding the promotion of wound healing by hucMSC-Exosomes, the molecular mechanisms underlying the actions of different cell populations and the interactions between cells have not been consistent in reports (7–9). This is likely due to the diversity of cells involved in wound repair and the complexity of the immune microenvironment.

The development of single-cell RNA sequencing (scRNA-seq) technologies has made it possible to study the heterogeneity of cell populations. Single-cell sequencing can identify extreme variability in the expression levels of individual cells within a given cell type, elucidate the unique role of specific cell expression levels on the overall phenotype, and predict the interaction of different cellular ligand-receptor interactions. This study aimed to reveal the heterogeneity of neutrophil and macrophage cells during exosome-induced tissue repair by single-cell sequencing and to reveal the potential mechanisms of hucMSC-Exosome accelerated tissue repair by ligand-receptor interaction analysis.

## 2 Materials and methods

### 2.1 Preparation and culture of huc-MSCs

The isolation of human umbilical cord tissue-derived mesenchymal stromal cells (huc-MSCs) was executed using a previously established protocol, in which discarded umbilical cords were utilized as the source material (10). Specifically, the Wharton’s jelly was extracted from the cords post-removal of the vascular structures. The extracted samples were subsequently diced into one cubic millimeter segments, which were then dispersed into a culture medium comprising DMEM-F12 (manufactured by Gbico) supplemented with 10% fetal bovine serum (also from Gbico), 1% penicillin-streptomycin (also from Gbico) and 10 ng/ml basic fibroblast growth factor. The cells were incubated under conditions of 37°C and 5% CO^2^, and the medium was refreshed at an interval of twice per week. After a period of two weeks, cells with fibroblast-like morphological characteristics could be observed. As the cells reached a confluence level of 70-80%, they were sub-cultured. The study employed only huc-MSCs within passages 3 to 5.

### 2.2 Exosomes extraction and characterization

Upon subjecting pre-confluent huc-MSCs to incubation in a serum-free DMEM medium for a period of 48 hours, the supernatants were then collected and subjected to sequential centrifugation as previously outlined (10), utilizing a centrifugal force of 1,000×g for 10 minutes, 4,000×g for 20 minutes, and 10,000×g for 40 minutes. Subsequently, hucMSC-Exosomes were precipitated through the utilization of ultracentrifugation at a force of 100,000×g for 70 minutes at 4°C using a Beckman Coulter Optima L-80 XP ultracentrifuge. Finally, the resulting solution was re-suspended in PBS before undergoing filtration through a 0.22 µm filter.

### 2.3 Mice

In order to conduct *in vivo* assays of punch-biopsy wound healing, six-week-old C57/BL6j mice were procured from Charles River Laboratories in Beijing. These mice were maintained under standard conditions, characterized by a temperature range of 22-24°C and a light-dark cycle of 12 hours, with ad libitum access to food and water. The animals were randomly allocated to various experimental groups, and all protocols were granted ethical approval by the institutional committee of Medical School of Chinese People’s Liberation Army prior to initiation of the experiments involving animal subjects.

### 2.4 Punch-biopsy wound healing models

An intraperitoneal anesthetic solution of 1% pentobarbital sodium was used. Prior to the creation of 8-millimeter full-thickness cutaneous lesions in the midline of the shoulder region, the epidermal hairs of the mice were depilated. A sterile punch biopsy needle (Integra Miltex, Integra York, P.A., Inc.) was utilized for the infliction of the wounds. Twenty mice were randomly divided into two groups, which were subcutaneously injected with 100 micrograms of hucMSC-Exosomes or an equal volume of PBS around the wound edges at 4 injection sites, administered every 48 hours. On day 7, the periwound skin (mainly regenerated tissue) were excised for Single-cell RNA sequencing.

### 2.5 Tissue processing

Sterile PBS on ice was used to preserve skin tissue samples immediately after dissection. The samples were subsequently cleaned by PBS twice and cut into pieces sized 2-4 mm. Then tissues were processed following the instructions of the dissociation kit (Multi Tissue Dissociation Kit 1, Miltenyi Biotec, 130-110-201) to complete the dissociation. The single-cell suspension was passed through 70 and 40 μm cell strainers and centrifuged for 10 min, 500 × g at 4 °C. The red blood cell (RBC) lysis solution (130-094-183, Miltenyi Biotec) and Dead Cell Removal Kit (130-090-10, Miltenyi Biotec) were successively added to remove erythrocytes or dead cells. The process resulted in highly viable, typically ≥85%, single-cell suspensions. For immediate single-cell capture, the cells were resuspended in 0.04% Ultra-Pure BSA in PBS (Thermo Fisher Scientific) and concentration was adjusted to ≥10^6^ cells/ml.

### 2.6 Single-cell RNA sequencing

The utilization of microdroplets in conjunction with barcoded primer beads enabled the capture of individual cells through the implementation of a droplet-based ultra-high throughput system for parallel gene expression detection. The Chromium Next GEM Single Cell 3ʹ Kit v3.1, developed by 10x Genomics (Part Number 1000268), was employed in the creation of gene expression (GEX) libraries. The generation of gel bead-in-emulsions, as well as the reverse transcription (RT) of the single-cell suspensions, was accomplished through the utilization of the 10x Genomics Single cell chip (Chromium Next GEM Chip G Single Cell Kit, Part Number 1000120, and an additional Dual Index Kit TT Set A, Part Number 1000215) run on the Chromium Controller (Part Number 110211) developed by 10x Genomics. The generated cDNA was amplified in order to produce GEX libraries after the RT step, subsequently quantified through the use of Qubit 3.0 fluorometer (Life Technologies, Part Number 15387293) and assessed through the utilization of HS DNA chips (Agilent Technologies, Part Number 5067-4627) in combination with the 2100 Bioanalyzer (Agilent Technologies, Part Number G2939BA). The Novaseq 6000, developed by Illumina, was employed for massively parallel sequencing.

### 2.7 Data processing and analysis

Raw scRNA-seq datasets were aligned to the mm10 reference genome using the Cell Ranger V6.1.2 from 10X Genomics Inc. Downstream data processing and visualization were performed using Seurat V4.0.6 (11). The quality filtering on scRNA-seq data was performed by filtering cells expressing the lower number of genes (<200 genes), and genes only uniquely expressed in <3 cells. The percentage of mitochondrial genes was regressed during the subsequent normalization step. Potential doublets were identified by DoubletFinder V2.0.3 at a threshold of 10% and filtered out (12). The datasets were then merged and normalized using the SCTransform algorithm with the principal component parameter set to 30. Principal component analysis (PCA) was followed by Uniform Manifold Approximation and Projection (UMAP) analysis to cluster cells with similar gene expression patterns together. Well-established cell marker genes are then used to annotate each cluster. Immune cells (Ptprc+) were computationally separated as described by Vu et al. (13). Non-parametric Wilcoxon Rank Sum test (p Adjusted value = 0.01, Fold Change = 1.2) was used to identify differences between treatment groups.

### 2.8 Cellular trajectory and RNA velocity analysis

Cellular transcriptomic profiles were used as input for Slingshot Package V2.4.0 (14) to predict differentiation trajectory and pseudotime data. The Gene expression matrix of each cell was fed into Tradeseq Package V1.10.0 to calculate the imbalance score, topology test value, and the subsequent differential trajectory between treatment and control (15). P value < 0.05 was considered significant for the topology test to determine whether treatment and control share a common trajectory. The unsupervised RNA velocity analysis was done with ScVelo (16).

### 2.9 Pathways and systems biology analysis

Gene Set Enrichment Analysis (GSEA) was performed to better understand the changes in biological pathways with treatment. Genes were sorted based on average log fold change along the trajectories. The result was fed to Fgsea package V1.22.0 (17) in combination with pathway data from MSigDB V7.5.1 (18) to generate a list of relevant enriched pathways. The significance of each biological pathway was determined by the permutation test. The detected biological pathways with p-value <0.05 were considered significant. Kyoto Encyclopedia of Genes and Genomes (KEGG) analysis was performed. Differentially expressed genes were identified with the non-parametric Wilcoxon Rank Sum test (P-value <0.01 and Fold Change >2). R package clusterProfiler V4.4.4 was used to identify influenced pathways (19).

### 2.10 Real-time qPCR

Total RNA was extracted using Trizol^®^ reagent (Invitrogen) from tissues and cells. The cDNA was obtained using Prime Script™ RT Master Mix (TaKaRa Bio, RR036A). The qRT-PCR was performed according to the instructions for Power SYBR Green PCR Master Mix (Applied Biosystems, USA). Relative expression levels were calculated using the comparative threshold cycle (2^-ΔΔCt^) method (20). Statistical significance was determined by two-tail unpaired t-tests, with p-values smaller than 0.05 considered significant. The GAPDH was employed as the internal control. The primer sequence is as follows:

*CCL6*: CACCAGTGGTGGGTGCATCAAG/GTGCTTAGGCACCTCTGAACTC

*CXCL3*: TGAGACCATCCAGAGCTTGACG/CCTTGGGGGTTGAGGCAAACTT

*IL1RN*: TGTGCCTGTCTTGTGCCAAGTC/GCCTTTCTCAGAGCGGATGAAG

*CXCL9*: CTGTTCCTGCATCAGCACCAAC/TGAACTCCATTCTTCAGTGTAGCA

*CXCL10*: GGTGAGAAGAGATGTCTGAATCC/GTCCATCCTTGGAAGCACTGCA

*CXCL16*: CCTATGTGCTGTGCAAGAGGAG/CTGGGCAACATAGAGTCCGTCT

### 2.11 ELISA

After the tissues are shredded and homogenized, the detection of cytokines (IL-4, IL-13, IL-17A, and IL-33) from tissue lysates was performed *via* ELISA according to the manufacturer’s (R&D Systems) instructions.

### 2.12 Ligand and receptor-based cell interaction analysis

NicheNetR V1.1.0, a method combining gene expression data with existing knowledge on ligand-to-target signaling paths to predict ligand-receptor interactions, was used to investigate alterations in the relationship among different cell types caused by exposure to hucMSC-Exosomes (21). Differential gene expressions are combined with NicheNetR ligand-target weights to compute the Pearson correlation coefficient between ligand-receptor interaction. Ligand-receptor interactions with an expression of less than 10% were filtered out.

### 2.13 Histological analysis

Skin samples were fixed with 4% paraformaldehyde (PFA) to preserve tissue morphology. The fixed samples were subsequently embedded in paraffin and cut into 5-µm sections for histological analysis. Hematoxylin and eosin (H&E) and Masson Trichrome staining were performed using routine procedures.

### 2.14 Immunofluorescence staining and imaging

Skin samples were obtained from mice and subsequently preserved in 4% PFA prior to sectioning into 5-μm slices. These sections were subjected to staining with antibodies against F480 (Cell Signaling Technology, 1:1000), Arg1 (Proteintech, 1:400), LY6G (ThermoFisher, 1:1000), BCL-2 (Bioss,1:200), and SLC2A3 (Proteintech, 1:100). Subsequently, the fluorescent signals of these markers were visualized and captured using a fluorescent inverted microscope.

### 2.15 Exosome uptake assay

HucMSC-Exosomes were mixed with PKH26 and Diluent C (MINI26, Sigma Aldrich) for a duration of 5 minutes. To stop the reaction, 0.5% FBS was added, and the exosomes were then treated with a sucrose solution and subjected to ultracentrifugation in the dark at 120,000 × g for 90 minutes. Labeled hucMSC-Exosomes or PKH26-labeled control were then administered through subdermal injection into mouse periwound tissue, and the samples were collected after 4 and 24 hours. Subsequently, the nuclei of the cells were stained with 4’,6-diamidino-2phenylindole (DAPI; Leagene, China), and images were obtained using a confocal microscope.

## 3 Results

### 3.1 HucMSC-exosomes induce functional changes in neutrophils

**Figure 1:**
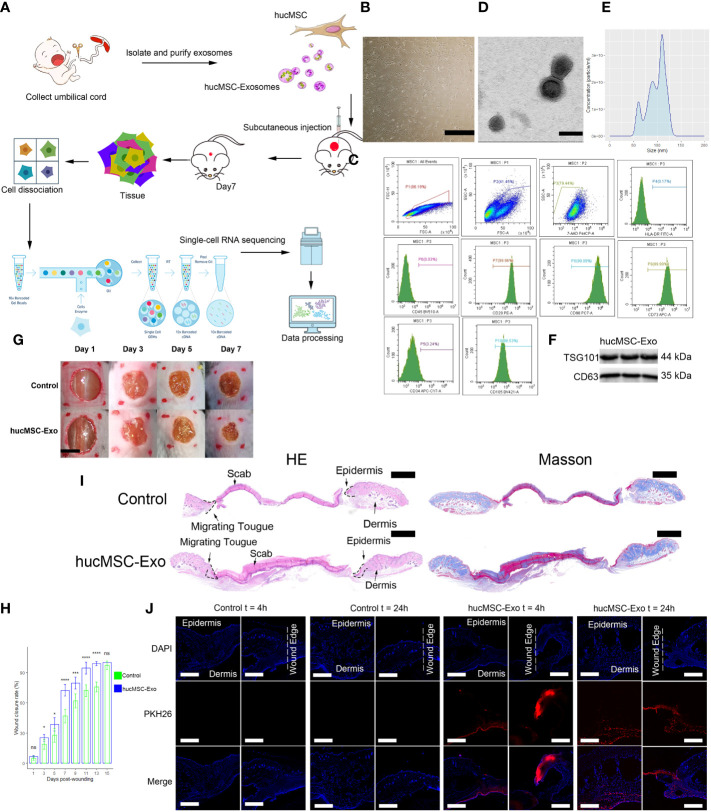
HucMSC-Exosomes accelerate wound healing. **(A)** Schematic overview of the study design. **(B)** Microscope image of huc-MSCs. The scale bar is 500 µm. **(C)** Flow cytometry analysis of HLA, CD29, CD34, CD45, CD73, CD90 and CD105 expression on the huc-MSC surface. **(D)** Transmission electron microscopy image of hucMSC-Exosomes. Scale bar, 100 nm. **(E)** Density plot of hucMSC-Exosomes. **(F)** Western blot of hucMSC-Exosome markers **(G)** Representative images of wound closure process of hucMSC-Exosomes treatment and PBS control. The scale bar is 4 mm. **(H)** Wound closure rate of hucMSC-Exosomes treatment and PBS control group. Two-tailed unpaired t-test (n=6). Error bar: mean standard deviation. ns, not significant, *p < 0.05, ***p < 0.001, ****p < 0.0001. **(I)** H & E and Masson staining of wounds seven days after injury. Scale bar = 1mm. **(J)** Uptake of hucMSC-Exosome by skin wound *in vivo*. Scale bar, 500μm.

The isolation and culture of huc-MSCs was performed in adherence to established methods (**Figure 1A**), resulting in the generation of a monolayer of spindle-shaped cells (**Figure 1B**). The isolated huc-MSCs were verified for the expression of mesenchymal stem cell markers (CD90 and CD105) and the absence of hematopoietic stem cell markers (CD34 and CD45) *via* flow cytometry analysis (**Figure 1C**). Transmission electron microscopy revealed the exosomes to be spherical in shape, with a hypodense center and typical saucer-like structure enclosed by clear membranes (**Figure 1D**). The particle size distribution of the exosomes was observed to range from 60 to 150 nm in diameter, and the zeta potential was measured to be -22 ± 2.9 mV (n=3) (**Figure 1E**). Furthermore, western blot analysis confirmed the expression of TSG101 and CD63 all of which are widely recognized as biomarkers of exosomes (**Figure 1F**). Compared to the control group (PBS injection), mice in the hucMSC-Exosomes intervention group had significantly faster healing rates (**Figures 1G, H**). Increased re-epithelialization were visible in H&E staining with higher collagen content detected through Masson staining (**Figure 1I**) (22). The absorption of hucMSC-Exosomes was visualized *via in vivo* exosome uptake assay at 4h and 24h (**Figure 1J**).

Neutrophils and macrophages play a vital role in the initial phase of wound healing, helping to clear the wound of bacteria and debris and laying the foundation for subsequent tissue repair. To determine the heterogeneity of neutrophils and macrophages in hucMSC-Exosome-induced wound repair, we collected periwound skin from mice on postoperative day (POD) 7 (the most significant time point for difference in healing rate) for scRNA-seq analysis (**Figure 1H**) (22).

**Figure 2:**
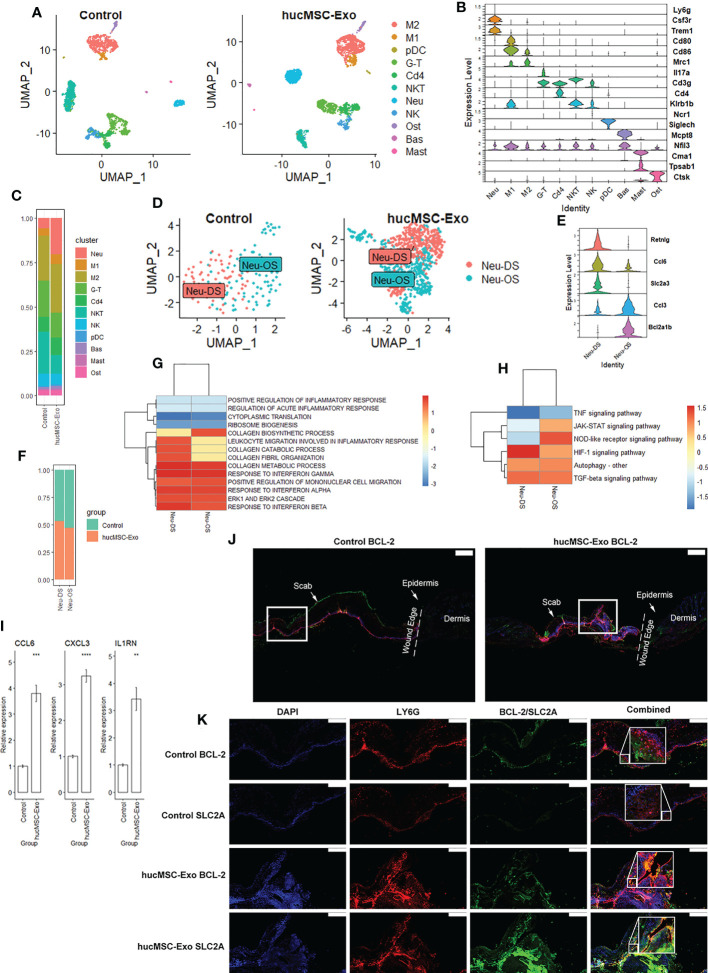
HucMSC-Exosomes induce functional changes in neutrophils. **(A)** 2D scatterplot based on UMAP reduction of immune cell coordinates. **(B)** Stacked violin plot of immune cell annotation markers. **(C)** Stacked bar plot of immune cell abundance ratio. **(D)** 2D scatterplot based on UMAP reduction of neutrophil coordinates. **(E)** Stacked violin plot of neutrophil subcluster annotation markers **(F)** Stacked bar plot of neutrophil subcluster abundance ratio. **(G)** GSEA Heatmap of neutrophil differentially expressed genes (DEGs) following hucMSC-Exosomes exposure **(H)** KEGG Heatmap of neutrophil DEGs following hucMSC-Exosomes exposure. **(I)** Quantitative RT-PCR analysis of the *Ccl6*, *Cxcl3*, and *Il1rn* mRNA expressions in peri-wound samples. Two-tailed unpaired t-test (n = 6). Error bar: mean standard error. GAPDH was used as the reference gene. **p < 0.01, ***p < 0.001, ****p < 0.0001. **(J)** Merged immunofluorescence staining with BCL-2 (green), LY6G antibody (red) and nuclear staining (DAPI). The scale bar is 500 µm. White square indicate the region of **(K)**. **(K)** Immunofluorescence staining with BCL-2 or SLC2A antibody (green) and LY6G antibody (red). Nuclear staining (DAPI) and merged images are also shown in the diagram. The scale bar is 200 µm.

We analyzed samples from two mice randomly chosen from six in the control group (cell count = 12969, 8804) and two mice randomly chosen from six in the hucMSC-Exosomes group (cell count = 10928, 9838) on POD 7. ScRNA-seq data analysis was performed using Seurat version 4.0.6. During the quality control process, we eliminated cells expressing gene counts >3000 or <200, and cells with >10% mitochondrial genes. DoubletFinder V2.0.3 was used to remove doublets (12). Subsequently, UMAP coordinates were calculated for each cell, and cells with similar expression profiles were clustered together. 11 immune cell types were identified *via* well-established marker genes (**Figure 2A**). These are, in descending order of abundance, Neu: neutrophils (*LY6G*+, *CSF3R*+, *TREM1*+), M1:M1 macrophages (*CD80*+, *CD86*+), M2: M2 macrophages (*MRC1*+), G-T: γδ T cells (*IL17A*+, *CD3G*+), NKT: NKT cells (*CD3G*+, *KLRB1B*+), Cd4: Cd4+ T cells (*CD4*+, *CD3G*+), NK: NK cells (*NCR1*+), Ost: osteoclast-like macrophages (*CTSK*+, *CD68*+), Bas: basophils (*MCPT8*+, *NFIL3*+), pDC: plasmacytoid dendritic cells (*SIGLECH*+), Mast: mast cells (*CMA1*+, *TPSAB1*+) (**Figure 2B**). Abundance ratios were calculated, the hucMSC-Exosomes group had significantly higher M1 macrophages (215 (1.0%) vs 76 (0.4%), p < 0.00001, chi-square test), M2 macrophages (1231 (5.9%) vs 670 (3.1%), p < 0.00001, chi-square test), and neutrophils (930 (4.5%) vs 157 (0.7%), p < 0.00001, chi-square test) (**Figure 2C**).

Upon tissue injury, neutrophils are recruited to the site of the wound where they phagocytose and kill invading microorganisms. They also release various cytokines and enzymes that contribute to the inflammatory response and stimulate the proliferation of other immune cells. Neutrophils in our dataset can be further divided into 2 subclusters: Neu-DS: migrated neutrophils and Neu-OS: proliferated neutrophils (**Figure 2D**). *RETNLG*, a gene suppressed in neutrophils in inflamed tissue (23), is the marker specific for the migrated neutrophils. Another marker for migrated neutrophils is *SLC2A3*, a gene associated with epithelial-mesenchymal transition (EMT) pathways (24). Conversely, *BCL2A1B*, an anti-apoptosis gene for neutrophils (25), can be a marker for proliferating neutrophils. Migrated neutrophils had increased expression of *CCL6* while proliferating neutrophils produced more *CCL3* (**Figure 2E**). Subtype ratios were similar between groups (p=0.16, chi-square test) (**Figure 2F**). The top expressed genes for each neutrophil subtype were illustrated in (**Supplementary Figure 1A**).

We then investigated the functional differences between the two subtypes. GSEA showed that migrated neutrophil favors biological processes such as neutrophil migration, chemotaxis, and extravasation while proliferated neutrophils favor cytoplasmic translation and ribosomal biogenesis (**Supplementary Figure 1B**). KEGG analysis showed upregulation of JAK-STAT and HIF-1 signaling in migrated neutrophils and NOD and TNF signaling in proliferated neutrophils (**Supplementary Figure 1C**).

When under the influence of hucMSC-Exosomes, migrated neutrophils reduced ribosomal gene expressions (*RPS27A*, *RPS19*), upregulated chemokines expression (*CXCL3*, *CCL4*), and *CD33* expression (**Supplementary Figure 1D**). *CD33* constitutively inhibits the production of pro-inflammatory cytokines such as TNF-α, IL-1β, and IL-8 (26). Biological processes such as migration as well as responses to interferon alpha, beta, and gamma were elevated with decreased inflammation response after hucMSC-Exosome treatments (**Figure 2G**). NOD, TGF-β, and HIF-1 signaling were stimulated while JAK-STAT and TNF signaling were suppressed (**Figure 2H**). Similarly, proliferated neutrophils also decreased expression of ribosomal genes (*RPL24*, *RPL32*) after hucMSC-Exosomes exposure, increased chemokines expression (*CCL6*, *CXCL3*) and *IL1RN* expression (**Supplementary Figure 1C**). *CXCL3* and *IL1RN* are critical for angiogenesis as well as collagen deposition (27, 28). Collagen metabolic process and ERK cascades were increased with decreased cytoplasmic translation and ribosomal biogenesis (**Figure 2G**). HIF-1 and autophagy were upregulated while IL17 and TNF signaling were downregulated. Elevated expression of *CCL6*, *CXCL3*, and *IL1RN* was then confirmed *via* qRT-PCR on the sample (**Figure 2I**).

In order to assess neutrophil distribution within the wound site, we employed immunological co-staining techniques. The treatment with hucMSC-Exosomes was accompanied by a marked increase in neutrophil density, as evidenced by the presence of *LY6G* on POD 7 (**Figures 2J, K**). In addition, we observed that *BCL2* and *SLC2A*, although both overlapping with *LY6G*, were localized to distinct regions within the wound site and exhibited minimal overlap. This suggests that these two markers are indicative of different subtypes of neutrophils. Specifically, *BCL2* was found to be concentrated at the upper edge of the wound and displayed a dispersed distribution pattern, while *SLC2A* was localized to the middle layer and displayed a more focused distribution pattern. These findings suggest that the neutrophils exhibiting these markers have distinct migratory behaviors within the wound site.

The temporal dynamics of gene expression during the process of wound healing can be analyzed through the application of RNA velocity and pseudotime analysis, offering insights into the molecular mechanisms underlying tissue repair. To date, there has been a lack of research examining the role of neutrophil activity in skin wound healing utilizing these techniques. We have employed a novel approach that combines trajectory analysis, RNA velocity analysis, and feature analysis within a single set of UMAP coordinates, leveraging recent advancements in single-cell analysis for improved synergy between the analyses. This approach has been endorsed by both the updated Monocle R package and the widely utilized Slingshot R package, both of which are commonly employed in the single-cell analysis (14, 29).

**Figure 3:**
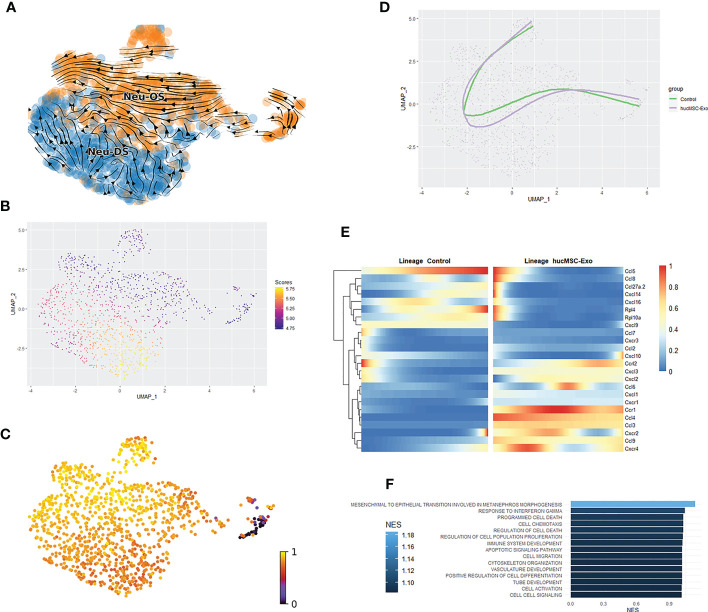
Pseudotime and RNA velocity profiles of neutrophils. **(A)** Scatterplot of neutrophils RNA velocity graph. **(B)** Scatterplot for imbalance score for neutrophils. Bright yellow regions indicate that nearby cells share the same condition. Deep blue regions mean that nearby cells are from a mix of the hucMSC-Exosomes group and the control group. **(C)** Latent time graph based on UMAP reduction of neutrophils; cells are colored based on latent time. **(D)** Scatter and trajectory plot based on UMAP reduction of neutrophils. **(E)** Heatmap of gene’s differential expression between conditions along the trajectory. **(F)** Barplot of the hucMSC-Exosomes groups vs the control group relevant GSEA elevated from differentially expressed genes between conditions along the trajectory.

The RNA velocity graph indicated the presence of two distinct flow patterns roughly equal in size, one highly organized flow stream starts from a differentiation point identified *via* the latent time graph. The organized nature of the cells in the stream signifies the presence of a complete differentiation path, therefore cells in the organized flow stream likely proliferated in the local wound. These are proliferated neutrophils. There is one disorganized flow stream with no clear start and end point. Likewise, disorganizations in the RNA velocity graph mean that most of the cells along the differentiation path were not captured in the sample, hence cells in the disorganized flow stream migrated to the wound site from various other locations (**Figure 3A**). These are migrated neutrophils.

A subsequent analysis of the imbalance score revealed a discrepancy in the disorganized region of the flow plot (**Figure 3B**). However, the Topology test was unable to confirm the existence of alternate trajectories for the hucMSC-Exosomes group (p = 0.43). The cell trajectories were calculated using the origin point identified through the latent time graph. The lineage of the proliferating neutrophils was successfully identified. The hucMSC-Exosomes treatment produced trajectories that were similar to those of the control group (**Figures 3C, D**).

During the process of differentiation, cellular expression of several markers integral to neutrophil function exhibited significant increases (**Figure 3E**). In the group treated with hucMSC-Exosomes, numerous chemokines displayed heightened expression at the onset of lineage development, including *CXCL16*, *CXCL9*, *CCL27A*, and *CXCL10*. However, expressions of these chemokines rapidly decreased along the differentiation trajectories to levels comparable to those observed in the control group. *CCL5* expression was observed in the early stages of the trajectory in the hucMSC-Exosomes group, and in the later stages in the control group. This suggests dynamic shifts in neutrophil priorities, as *CCL5* plays a crucial role in the recruitment of endothelial cell progenitors (30). *CXCL9*, *CXCL10*, and *CXCL16* have been shown to elicit type I immune responses and facilitate re-epithelialization, while *CCL27A* is critical for reducing inflammation (31, 32). GSEA for differential expression along the trajectory showed upregulation of wound healing-related aspects like cell migration, vasculature development, and positive regulation of cell differentiation (**Figure 3F**).

### 3.2 HucMSC-exosomes induce functional changes in macrophages

**Figure 4:**
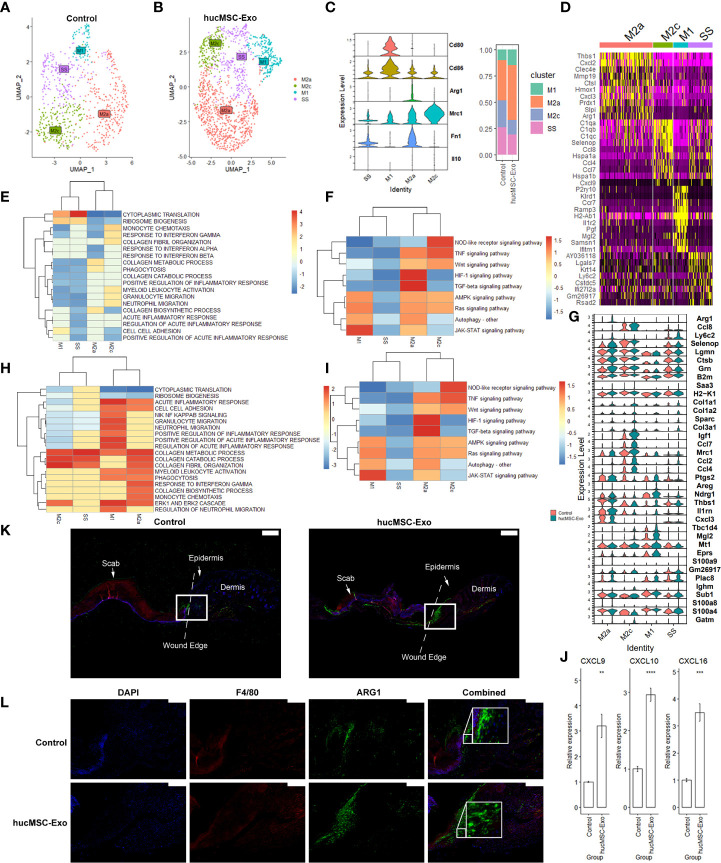
HucMSC-Exosomes induce functional changes in macrophages. **(A)** 2D scatterplot based on UMAP reduction of macrophage coordinates. **(B)** Stacked violin plot of macrophage subcluster annotation markers. **(C)** Stacked bar plot of macrophage subcluster abundance ratio. **(D)** Heatmap of top expressed marker of macrophage subclusters. **(E)** GSEA Heatmap of genes expressed by macrophages. **(F)** KEGG Heatmap of genes expressed by macrophages. **(G)** Stacked violin plot of top differentially expressed genes (DEGs) of macrophages following hucMSC-Exosomes exposure. **(H)** GSEA Heatmap of macrophage DEGs following hucMSC-Exosomes exposure. **(I)** KEGG Heatmap of macrophage DEGs following hucMSC-Exosomes exposure. **(J)** Quantitative RT-PCR analysis of the *Cxcl9*, *Cxcl10*, and *Cxcl16* mRNA expressions in peri-wound samples. Two-tailed unpaired t-test (n = 6). Error bar: mean standard error. GAPDH was used as the reference gene. **p < 0.01, ***p < 0.001, ****p < 0.0001. **(K)** Merged immunofluorescence staining with ARG1 antibody (green), F4/80 antibody (red) and nuclear staining (DAPI). The scale bar is 500 µm. White square indicate the region of **(L)**. **(L)** Immunofluorescence staining with ARG1 antibody (green) and F4/80 antibody (red). Nuclear staining (DAPI) and merged images are also shown in the diagram. The scale bar is 200 µm.

Macrophages are crucial for effective wound healing as they serve to clear debris, stimulate angiogenesis, and induce inflammation. The diverse subtypes of macrophages play a complex and dynamic role in the wound healing process, coordinating the inflammatory response and promoting tissue repair and remodeling. M2 Macrophages can be further subclustered into SS: steady-state macrophages, M2a: M2a macrophages, and M2c: M2c macrophages (**Figure 4A**). The M2a cluster was annotated based on its functional expression of *FN1*. While M2c was annotated based on the expression of *IL10* (**Figure 4B**). The hucMSC-Exosomes group had a higher ratio of M2a (745 (3.6%) vs 282 (1.3%), p < 0.00001, chi-square test) (**Figure 4C**). The top expressed genes for each macrophage subtype were illustrated in **Figure 4D**. Interestingly the IL10-rich cluster also coincides with focused expressions of *CCL2*, *CCL3*, *CCL4*, and *CCL5* (**Supplementary Figure 3**).

We subsequently examined the functional changes brought by hucMSC-Exosomes exposure. Compared to the rest of the macrophages, M1 polarized macrophages showed increased ribosomal biogenesis, cell-cell adhesion, and decreased migration and activation (**Figure 4E**). JAK-STAT and AMPK signaling were higher with lower TNF and HIF-1 signaling (**Figure 4F**). Following exposure to hucMSC-Exosomes, M1 macrophages had increased *VEGFA*, *IL1RN*, and *CXCL3* expression (**Figure 4G**, **Supplementary Figure 1E**), upregulated collagen metabolic process, migration, and ERK cascade (**Figure 4H**) as well as HIF-1, TNF signaling and autophagy (**Figure 4I**). Steady-state macrophages had higher cytoplasmic translation and ribosomal biogenesis with lower phagocytosis and inflammatory response (**Figure 4E**). Autophagy, HIF-1, TNF, and IL17 signaling were lower in steady-state macrophages (**Figure 4F**). Exposure to hucMSC-Exosomes lowered chemokine production (*CXCL2*, *CXCL9*) (**Figure 4G**, **Supplementary Figure 1E**), migration, inflammatory response (**Figure 4H**), TNF, HIF-1 and TGF-β signaling (**Figure 4I**). M2a macrophage subtype appears to focus on collagen metabolic process, migration, phagocytosis, autophagy, and HIF-1 signaling with lesser ribosomal biogenesis, interferon response, and IL17 signaling (**Figures 4E, F**). HucMSC-Exosomes triggered upregulation of *ARG1*, *LY6C2*, *CCL8* (**Figure 4G**), ECM remodeling, angiogenesis, migration, phagocytosis (**Figure 4H**), HIF-1, Ras, and IL17 signaling (**Figure 4I**). M2c macrophages favor chemotaxis, TNF, GPCR, IL17, and Wnt signaling pathways with less NF-κB, collagen biosynthesis, and HIF-1 signaling (**Figures 4E, F**). HucMSC-Exosomes simulated elevation of *CCL2*, *CCL4*, collagen catabolic process, and ECM receptor interaction (**Figures 4G–I**). Elevations of *CXCL9*, *CXCL10*, and *CXCL16* expression were verified with qRT-PCR (**Figure 4J**).

Immunological co-staining was utilized to evaluate the occurrence of periwound macrophages. Both the control group and the hucMSC-Exosomes group exhibited a dispersed pattern of macrophages (**Figures 4K, L**). The presence of macrophages was notable in both groups, but with significantly higher density in the hucMSC-Exosomes group as demonstrated by F4/80 staining. ARG1 staining, which visualizes M2 macrophages, revealed a dispersed pattern in the control group and a more focused, localized pattern in the hucMSC-Exosomes group at the base of the wound. These findings suggest that exposure to hucMSC-Exosomes may alter the migratory and functional profile of M2 macrophages.

**Figure 5:**
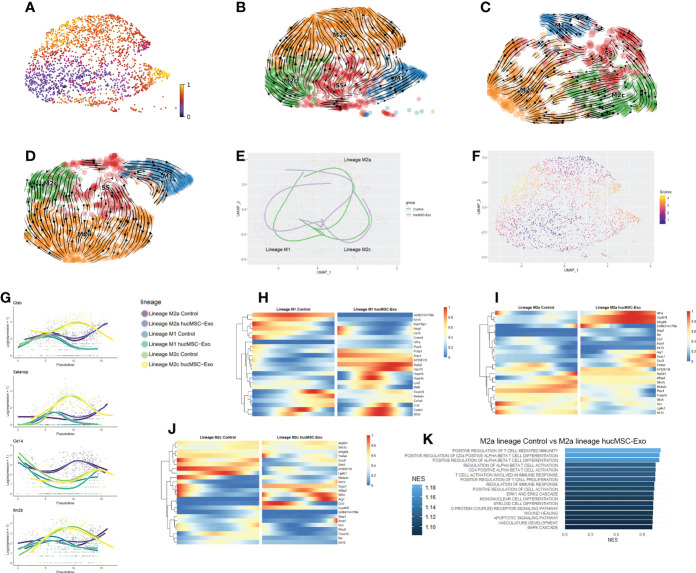
Pseudotime and RNA velocity profiles of macrophages **(A)** Latent time graph based on UMAP reduction of M1 and M2 macrophages; cells are colored based on latent time. **(B)** Scatterplot of macrophages RNA velocity graph. **(C)** Scatterplot of macrophages RNA velocity graph for the control group. **(D)** Scatterplot of macrophages RNA velocity graph for the hucMSC-Exosomes group. **(E)** Scatter and trajectory plot based on UMAP reduction of macrophages. **(F)** Scatterplot for imbalance score for macrophages. Bright yellow regions indicate that nearby cells share the same condition. Deep blue regions mean that nearby cells are from a mix of the hucMSC-Exosomes group and the control group. **(G)** Smoother plot of gene expression along trajectories for macrophages. **(H)** Heatmap of gene’s differential expression between conditions along the M1 trajectory. **(I)** Heatmap of gene’s differential expression between conditions along the M2a trajectory. **(J)** Heatmap of gene’s differential expression between conditions along the M2c trajectory. **(K)** Barplot of the hucMSC-Exosomes groups vs the control group GSEA biological pathways that are significantly (P value <0.05) elevated from differentially expressed genesalong the M2a trajectory.

Utilizing RNA velocity in conjunction with pseudotime analysis, we sought to investigate the functional changes in macrophages. By generating the unsupervised latent time graph from RNA velocity, we identified the center region of the UMAP scatterplot as the point of origin for macrophage differentiation (**Figure 5A**). Furthermore, we did not observe any distinctive expression of M1 or M2 markers in the origin point cluster. By combining latent time analysis with RNA velocity analysis of macrophages, we were able to identify three distinct endpoints of differentiation at the end of the M1, M2a, and M2c clusters (**Figure 5B**). The flow streams were highly organized and demonstrated a directional stream towards the regions of M1, M2a, and M2c. However, the M2c endpoint was absent from the control group flow stream plot, suggesting a shift in the priorities of differentiation (**Figures 5C, D**). We used the origin point from the latent time graph as input for subsequent pseudotime analysis, which generated three corresponding trajectories: the M1 Lineage, M2a Lineage, and M2c Lineage (**Figure 5E**). We then calculated the imbalance score between the control and hucMSC-Exosomes treatments to determine if hucMSC-Exosomes exposure affected the cellular trajectory (**Figure 5F**). A significant imbalance is visually apparent in the region bordering the M2a cluster and M2c cluster. The existence of an alternate trajectory for the hucMSC-Exosomes treatment was then verified using the Topology test (p <0.00001).

The elevated expression of *PLAC8* signifies that the steady-state macrophages were positioned in the lower wound area (**Supplementary Figures 1F, G**). Interestingly, only roughly half of the M2a macrophages along the M2a trajectory maintained the expression of *PLAC8*, while its expression was absent from M2c and M1 macrophages, indicating distinct migratory behavior across subcutaneous locations as the macrophage differentiates. Testing the lineages for early drivers of differentiation revealed *CTSB*, *SELENOP*, *CD14*, and *ITM2B* as potential candidates for differentiation drivers (**Figure 5G**). These genes have the strongest differences between lineages at the point of lineage divergence. *CTSB* has been associated with the memory immune response of macrophages against tumors (33).

*HIF1α* plays a vital role in the complex process of wound healing, by coordinating the expression of various genes involved in angiogenesis, immune cell activation, and inflammation. The expression of *HIF1α* increased along both lineage M2a and M2c but remained consistently low for the M1 lineage (**Figures 5H–J**). The *HIF1α* expression increased in the beginning in both the hucMSC-Exosomes group and control group along the M2a lineage, however, the *HIF1α* expression for the M2a control group decreased midway. Similarly, *HIF1α* expression was higher at the latter half of the M2c trajectory for the hucMSC-Exosomes group. Both M2a and M2c trajectories for the hucMSC-Exosomes group end with elevated *ARG1* expression, whereas their respective control trajectories plateaued. GSEA enrichment analysis shows an increase in the regulation of T cell activation and differentiation specifically for the M2a lineage in the hucMSC-Exosomes group (**Figure 5K**).

### 3.3 HucMSC-exosomes alter wound healing microenvironment through ligand-receptor interaction

The role of ligand-receptor interactions in wound healing is of paramount importance, as these interactions serve as the primary means of communication between cells and are essential for coordinating the various stages of tissue repair. In order to evaluate the alteration of the wound microenvironment from the perspective of ligand-receptor interactions, we utilized NicheNetR, a method that integrates gene expression data with existing knowledge on ligand-to-target signaling pathways to predict ligand-receptor interactions (21). The recent advances in NicheNetR methodology enabled us to perform a differential analysis of ligand-receptor interactions between treatment conditions.

**Figure 6:**
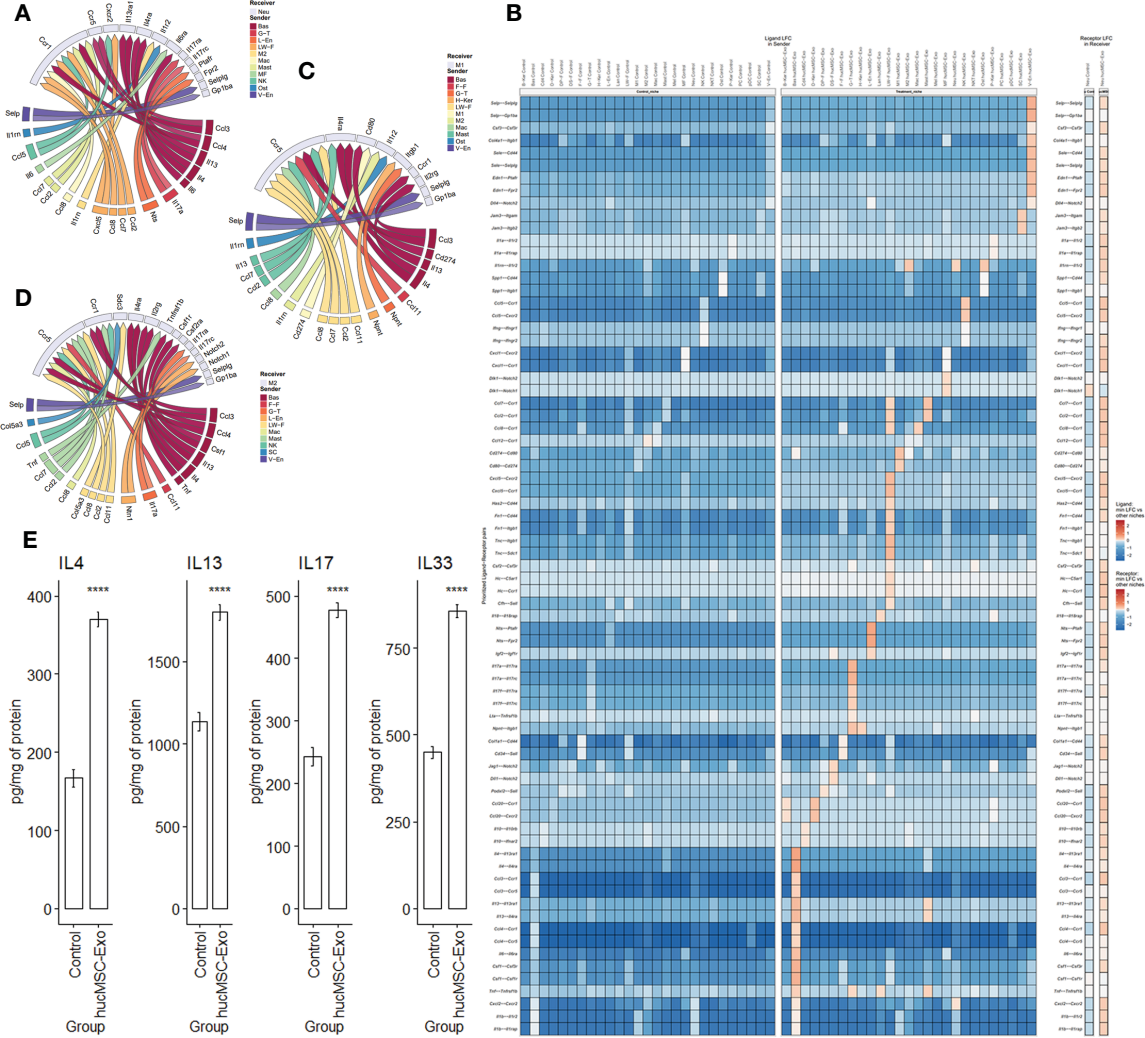
Ligand-receptor interactions after hucMSC-Exosomes exposure. **(A)** Circos plot of differential ligand-receptor interaction for neutrophils (receiver). **(B)** Heatmap illustrating ligand-receptor expression comparison between the control group and the hucMSC-Exosomes treatment group for neutrophils (receiver). **(C)** Circos plot of differential ligand-receptor interaction for M1 macrophages (receiver). **(D)** Circos plot of differential ligand-receptor interaction for M2 macrophages (receiver). **(E)** Cytokine protein levels for IL-4, IL-13, IL-17 and IL-33 measured by ELISA. Data are presented as mean and standard error. Unpaired two-tail t-test. ****p<0.00001.

The impact of hucMSC-Exosomes on basophils and neutrophils was marked by the interactions of *CCL3-CCR1*, *CCL4-CCR5*, *IL6-IL6RA*, and *IL13-IL13RA1* (**Figures 6A, B**). These interactions facilitated enhanced mobility and recruitment of neutrophils, as well as suppression of excessive neutrophil infiltration and consequent protection against unnecessary tissue damage (2, 34–36). In addition, hucMSC-Exosomes stimulated the lymphatic vessel endothelial cells to augment neutrophil phagocytosis through the *NTS-PTAFR* and *NTS-FPR2* interaction. (**Figure 6B**). After hucMSC-Exosomes treatment, basophils released more *CSF1*, *IL4*, *OSM*, and *LIF* (**Figures 6B–D**; **Supplementary Figures 2, 3**). *CSF1* regulates macrophage differentiation, proliferation, and survival (37). *IL4* suppresses macrophage pro-inflammatory mediators (38, 39). OSM stimulates M2 macrophage polarization (40). *LIF* stimulates macrophage infiltration (41). M2 macrophages secreted more *IL1RN* for competitive inhibition of *IL1A* and reduce neutrophil-associated inflammation (42). The significantly higher presence of IL-4, IL-13, IL-17, and IL-33 levels in samples was verified with ELISA (**Figure 6E**).

## 4 Discussion

The objective of our study was to utilize scRNA-Seq to examine the immune cell heterogeneity involved in wound healing in mice with full-thickness skin lesions that received hucMSC-Exosome treatment compared to controls (which received PBS injections). Our analysis identified a variety of immune cells, including macrophages, γδ T cells, neutrophils, NK cells, basophils, plasmacytoid dendritic cells, and mast cells. The proportions of most cell types were found to be within expected ranges according to previous literature (43, 44). Notably, we observed an elevation in the proportion of immune cells, particularly M2 macrophages and neutrophils, in wounds treated with hucMSC exosomes.

During the inflammatory phase, neutrophils are the first immune cells recruited to the wound to re-establish a temporary barrier against microbial invasion (45). Neutrophil infiltration is a prerequisite for the transition from inflammation to proliferation and is necessary for high-quality repair with activated neutrophils can also produce neutrophil extracellular traps to trap and eliminate exogenous pathogens (46). Our data showed the presence of two subtypes of neutrophils, namely, proliferated neutrophils and migrated neutrophils, each with unique chemokine expression and RNA velocity profile. Their presence was verified with immunofluorescence co-staining. *RETNLG* and *SLC2A3* are potential markers to identify migrated neutrophils of cutaneous wounds as *BCL2A1B* for proliferation neutrophils. This would help the identification and characterization of neutrophils in clinical and research settings. While both types of neutrophils serve critical functions, heterogeneity between migrated neutrophils and proliferating neutrophils in core neutrophil function was observed. The specialized function of migrated neutrophils at wound sites is characterized by enhanced migration, chemotaxis, extravasation, and upregulated HIF-1 signaling. These cells also favor the production of *CCL6*, which serves to recruit additional macrophages, thereby contributing to the increase in macrophage numbers at the site. In contrast, proliferated neutrophils stimulate tissue renewal through increased translation, proliferation, and NOD signaling. These cells preferentially produce *CCL3*, which stimulates angiogenesis. When introduced to the system, hucMSC-Exosomes elicit distinct responses in the two subtypes of neutrophils. Proliferated neutrophils further promote tissue regeneration through increased angiogenesis and collagen deposition, while the introduction of hucMSC-Exosomes leads to a reduction in inflammation in migrated neutrophils, thereby protecting against excessive tissue damage (27, 47). Activated neutrophils can release cytokines to prolong and amplify neutrophil infiltration (48). Therefore, it is interesting how recruited neutrophils facilitate the recruitment of other immune cells whereas proliferating neutrophils stimulate proliferation. Utilizing recent advances to chart RNA velocity, trajectory, and expression profile on a single set of cellular UMAP coordinates, we identified unique RNA velocity patterns that differentiate the two neutrophil subtypes. Continuous and organized streams in RNA velocity indicate that a significant portion of the cellular lifecycle was captured within the sample, which likely means the cells were proliferated from a common local source. The minimal presence of neutrophils in normal skin tissue suggests that the likely source of these cells is infiltrated, first responders. This finding suggests the existence of a temporal threshold that dictates the genotype of neutrophils. It is possible that either the heterogeneity of neutrophils from various sources or the alteration of the wound microenvironment determines the transition of migrated neutrophils into proliferated neutrophils. The present study introduces a novel annotation technique that utilizes RNA velocity profiles to characterize clusters. This method has proven efficacious in the classification of subclusters and the characterization of cellular origins. It is worth noting that, although neutrophil numbers exhibited the most substantial increase, the predicted trajectories were relatively similar between the control group and the hucMSC-Exosomes group. This finding suggests that neutrophil differentiation and infiltration are more resistant to qualitative changes than quantitative changes in response to the substantial microenvironmental modifications induced by hucMSC-Exosomes.

Neutrophils possess a plethora of surface receptors, including GPCR, pattern recognition receptors, Fc receptors, and other receptors that aid in the detection of injury signals such as chemokines, damage-associated molecular patterns, and hydrogen peroxide released from damaged tissue (2). Among the multitude of potential interactions, ligand-receptor analysis has revealed basophils as the primary mediators of the effect of human umbilical cord mesenchymal stem cell-derived exosomes, with *CCL3*, *IL6*, and *IL13* identified as the core driving ligands. The transcriptomic profiles of hucMSC-Exosomes were analyzed by Luo, T., et al., the study showed the presence of MIF in the exosomes which contribute to the increased CCL3-CCR1, CCL4-CCR5, and IL6-IL6RA ligand-receptor interaction, leading to enhanced cellular proliferation and survival (49–52). Our findings are in line with these results, which demonstrated the proliferation of neutrophils at the wound site. Furthermore, the anti-inflammatory effects of M2 macrophages extend to neutrophils *via* the release of IL1RN. However, it must be noted that only a small proportion of identified ligand-receptor interactions have been documented with respect to their impact on wound healing. Thus, further characterization of these interactions is necessary in order to fully comprehend the effect of human umbilical cord mesenchymal stem cell-derived exosomes on the inflammatory response.

Macrophages are also important for wound healing and regeneration. 2-3 days after skin damage, macrophages from local tissue and bone marrow accumulate in the wound. Studies have shown that wound healing is delayed in macrophage depletion, as evidenced by reduced angiogenesis and collagen deposition, and reduced growth factor release (53–56). In contrast, increasing the number of macrophages in the wound can significantly accelerate wound healing, as observed in our samples (57). M1 macrophages are generally considered to be pro-inflammatory macrophages, characterized by the expression of TNF-α, IL-6, and IL-1B, capable of recognizing pathogens and phagocytosing them, as well as promoting MMP synthesis and degrading ECM (1, 58). In the present investigation, the selected temporal parameter for obtaining samples was POD 7, characterized by the continued proliferation of various cells and the coexistence of inflammation and proliferation, as well as the remarkable acceleration of the healing rate in the experimental group compared to the control group. Our observations are in line with multiple studies, which collectively indicate that hucMSC-Exosomes play a preeminent role at this particular time point (59).

As the inflammation subsides, macrophages transform into an anti-inflammatory phenotype, M2 macrophages, which primarily promote angiogenesis as well as ECM deposition (60). Chen et al. (61) reported that hucMSC-Exosomes can facilitate M2 macrophage polarization with increase Arg1 expression. In line with these results, our investigation revealed increased M2 macrophage number and enhanced expression of genes associated with the anti-inflammatory properties of M2 macrophages. Additionally, our trajectory analysis of macrophage differentiation points to a reinforcement of the M2a macrophage subtype.

The highly organized and structured nature of the macrophage RNA Velocity graph, particularly when juxtaposed with that of neutrophils, suggests that the polarization event of macrophages is likely to be localized and emanating from a single, consistent cell source. Through the application of predictive modeling techniques, we hypothesized trajectories of steady-state macrophages differentiating into M1, M2a, and M2c phenotypes. In particular, M2a macrophages have been shown to possess the ability to promote the conversion of fibroblasts into myofibroblasts, thereby increasing collagen deposition, as well as the capacity to transform themselves into fibroblasts and deposit ECM components (62). On the other hand, M2c macrophages exhibit a fibrinolytic phenotype and are typically observed following re-epithelialization to phagocytose excess neutrophils, stromal cells, and other debris (63, 64). The variance in RNA velocity between treatment groups suggests that M2c macrophages may exhibit further differentiation into M2a macrophages in the control group while being more inclined to remain as M2c macrophages in the hucMSC-Exosomes group.

Predicted trajectories are observed to diverge significantly between conditions, thus indicating that macrophages differentiate differently when exposed to hucMSC-Exosomes. Early differential markers such as *CTSB*, *SELENOP*, and *CD14* have been identified. Given that polarizations of M2a, M2c, and M1 macrophages are induced by elements that are enriched within the cutaneous wound site, it is not surprising that the presence of hucMSC-Exosomes resulted in a divergence in trajectory for the macrophages. M1, M2a, and M2c differentiation trajectories exhibit distinct behavior while sharing a common starting point. This phenomenon is further compounded by the fact that the hucMSC-Exosomes group and the control group do not share trajectories.

Functional analysis demonstrated that exposure to hucMSC-Exosomes led to a decrease in the inflammatory capabilities of M1 macrophages, while both M2a and M2c macrophages acquired an enhanced anti-inflammatory capability along their respective trajectories. Furthermore, shifts in positional markers such as *PLAC8* suggest that macrophages exhibit a preference for distinct locations under the influence of hucMSC-Exosomes, which may be explained by altered ligand-receptor interactions of target cells.

A study on the proteomics of hucMSC-Exosomes is consistent with our research results on macrophage ligand-receptor interactions, i.e., the dataset (PXD020948, ProteomeXchange Consortium) listed the presence of S100A8/A9 and TGF-β in hucMSC-Exosomes (65). S100A8/A9 could directly result in elevated interaction between IL6-IL6RA and may stimulate TNF-α, leading to increased expressions of LIF and IL1RN for enhanced cellular proliferation and differentiation (66–70). TGF-β might partially be accountable for the heightened interaction between IL13-IL13RA1 and the expression of CSF1, supporting macrophage differentiation and debris clearance (71, 72). It is also consistent with our results which showed increased M2 macrophages polarization and activity. Through a functional enrichment analysis of DEGs in macrophages, our investigation revealed that the activation of the Wnt signaling pathway was notably augmented following treatment with hucMSC-Exosomes, alongside the amplification of supplementary pathways such as HIF-1. It is noteworthy that the Wnt pathway holds a pivotal role in modulating various cellular processes, including but not limited to the regulation of cell proliferation and migration, thus establishing its relevance in skin cell homeostasis. As indicated in previous studies, the nuclear translocation of β-catenin, a downstream effector of the Wnt pathway, promotes the aforementioned phenotypes in skin cells (73). In addition, upregulated TLR4 is believed to be involved in hucMSC-derived exosome-induced M2 macrophage inflammation and repair (74). Similar to reported hucMSC-Exosomes’ effect on liver, we also detected the elevation of ERK pathway in neutrophils, its promotion of cell survival may be the cause of increase neutrophils (75).However, it is important to note that hucMSC-Exosomes consist of hundreds of proteins, RNAs, and miRNAs, which collectively result in multifaceted effects from complex combinations of multiple molecules (7, 49, 65, 76–78). Therefore, it is improbable that a single bioactive molecule *in vivo* could replicate the effects of hucMSC-Exosomes in the microenvironments of wounds. The isolation and identification of specific molecules’ functional characteristics would be a significant undertaking and remain a key objective of our future studies. Currently, it is still quite difficult to simulate and verify the functions of specific molecules in the microenvironment of a wound *in vitro*, which will also be a goal for our future research.The healing of wounds is a complex biological process that involves the coordinated interactions between cytokines and various cells. HucMSC-Exosomes may directly release or induce the secretion of certain cytokines to promote wound healing (79). Interleukins (ILs) are a family of cytokines that play a key role in the process of wound healing. ILs are involved in a variety of immune and inflammatory responses, including the recruitment of immune cells to the site of injury. In the context of wound healing, ILs have been shown to promote the proliferation and migration of various cell types, including fibroblasts, keratinocytes, and endothelial cells. We observed elevated expression of various ILs including IL-4, IL-13, IL-17, and IL-33 with the result verified with ELISA. IL-4 has been shown to inhibit the production of pro-inflammatory cytokines, such as tumor necrosis factor-alpha (TNF-alpha) and interleukin-1 beta (IL-1 beta), which can delay wound healing if present in excess (80). IL-13 has similar effects to IL-4 with the additional effect of promoting the differentiation of fibroblasts into myofibroblasts, a specialized cell type that is responsible for wound closure by contraction (81). IL-17 has also been shown to enhance the production of collagen and stimulate the migration of neutrophils (82). IL-33 enhances the activation and recruitment of immune cells, such as neutrophils and eosinophils (83). A recent report has identified elevated levels of IL-8 in keratinocytes in response to exosome exposure at the wound site (84). However, no studies have yet characterized the depth and breadth of interleukins’ involvement in exosome treatments. This phenomenon is most efficiently evaluated through analysis of single-cell level transcriptomic profiles on tens of thousands of cells. The association between hucMSC-Exosomes and ILs has implications beyond wound healing, as it may have potential applications in cancer treatment as well (79). Revealing the possible role of IL in the microenvironment of wound healing may help in the development of targeted and engineered extracellular vesicles for use in the treatment of wound repair.

Through the utilization of a novel and high-throughput sequencing methodology known as single-cell sequencing, this study unveils the distinctive cellular factors and gene expression profiles that drive wound healing subsequent to full-layer skin damage in neutrophils and macrophages stimulated by the hucMSC-Exosomes. Moreover, this work elucidates the global impact on the expression level of such cells. Infiltration of the wound by neutrophils and macrophages is imperative to eliminate pathogens and synergistically regulate the healing process, with each phase necessitating coordination by a multitude of bioactive molecules through mediated cellular interactions. We provide a detailed description of the cellular fate of neutrophils and macrophages under the influence of hucMSC-Exosomes at the single-cell level, highlighting the upregulation of novel targets such as OSM, LIF and CSF1 etc. These molecules represent promising targets for exosome modification, thereby unveiling new prospects for future exosome application in wound repair and tissue regeneration. Additionally, we anticipate that the analysis of ligand-receptor interactions and pseudotime prediction between distinct cells will facilitate the prediction of potential mechanisms by which hucMSC-Exosomes promote wound healing, thus providing new ways for targeted therapy and engineered exosomes for wound repair.

## Data availability statement

The datasets presented in this study can be found in online repositories. The names of the repository/repositories and accession number(s) can be found below: GSE224491 (GEO).

## Ethics statement

The animal study was reviewed and approved by institutional committee of the PLA School of Medicine.

## Author contributions

YYL and RY contributed to conception and design of the study. MZ, KL, LW organized the database. YYL performed the statistical analysis. YYL wrote the first draft of the manuscript. YL, HC, DS, JL, and XZ wrote sections of the manuscript. All authors contributed to the article and approved the submitted version.
