# Corrigendum: Human umbilical cord mesenchymal stem cell-derived exosomes promote murine skin wound healing by neutrophil and macrophage modulations revealed by single-cell RNA sequencing

**DOI:** 10.3389/fimmu.2023.1181215

**Published:** 2023-03-14

**Authors:** Yuanyuan Liu, Mingwang Zhang, Yong Liao, Hongbo Chen, Dandan Su, Yuandong Tao, Jiangbo Li, Kai Luo, Lihua Wu, Xingyue Zhang, Rongya Yang

**Affiliations:** ^1^ Medical School of Chinese People’s Liberation Army, Beijing, China; ^2^ Department of Dermatology, the Seventh Medical Center of Chinese People’s Liberation Army (PLA) General Hospital, Beijing, China; ^3^ Department of Dermatology, Southwest Hospital, Army Medical University, Chongqing, China; ^4^ School of Pharmaceutical Sciences, Sun Yat-sen University, Shenzhen, China; ^5^ Department of Pediatric Urology, the Seventh Medical Center of Chinese People’s Liberation Army (PLA) General Hospital, Beijing, China; ^6^ Bioinformatics Center of Academy of Military Medical Sciences, Beijing, China; ^7^ Biomedical Treatment Center, the Seventh Medical Center of Chinese People’s Liberation Army (PLA) General Hospital, Beijing, China

**Keywords:** exosomes, wound healing, single-cell RNA sequencing, cellular heterogeneity, neutrophils, macrophages

In the published article, there was an error in the legend for **Figure 1** as published. The legend for **Figure 1A** was missing and the legend for **Figures 1A–F** was misaligned. The corrected legend appears below.

**Figure 1:**
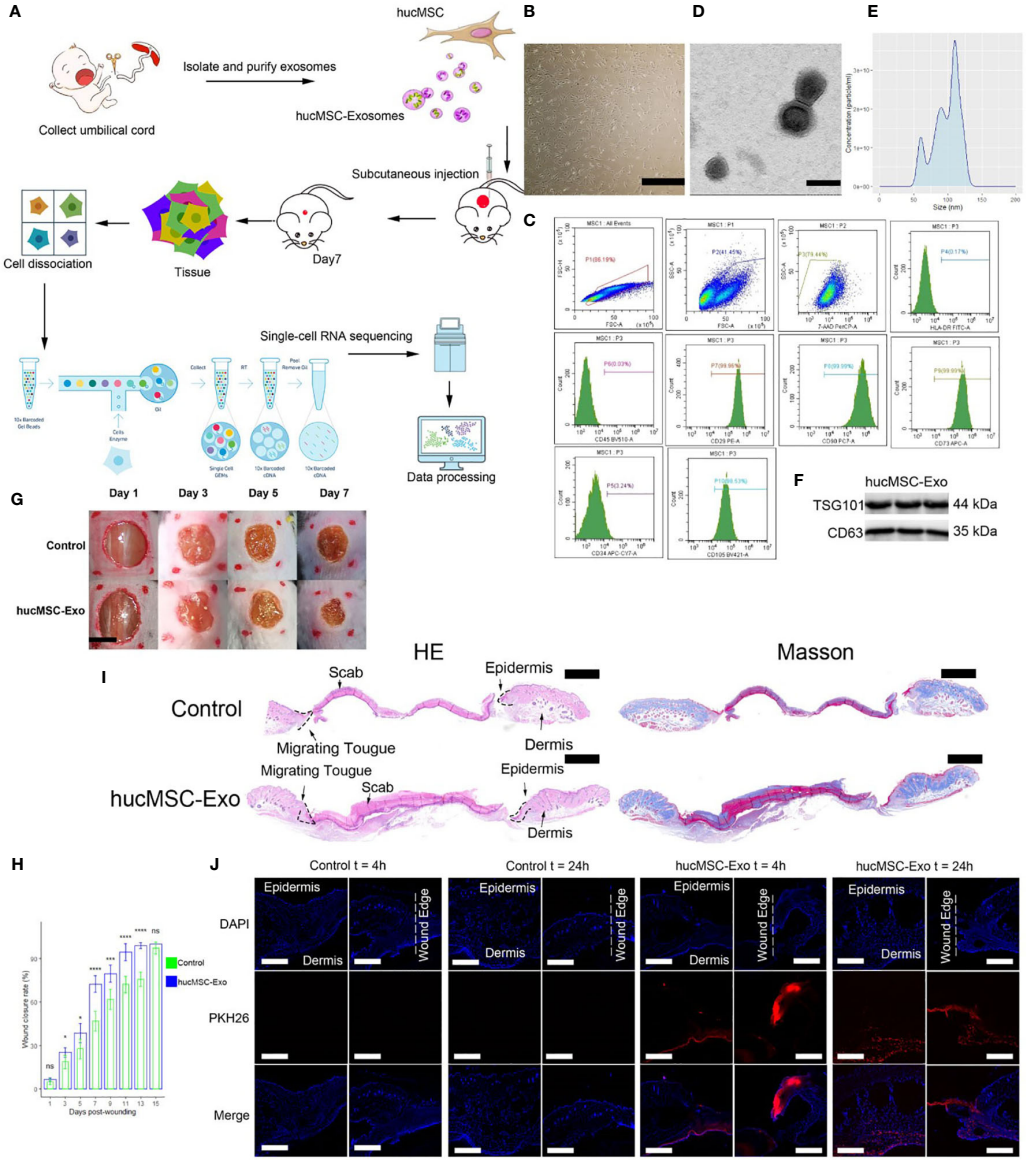
HucMSC-Exosomes accelerate wound healing. **(A)** Schematic overview of the study design. **(B)** Microscope image of huc-MSCs. The scale bar is 500 µm. **(C)** Flow cytometry analysis of HLA, CD29, CD34, CD45, CD73, CD90 and CD105 expression on the huc-MSC surface. **(D)** Transmission electron microscopy image of hucMSC-Exosomes. Scale bar, 100 nm. **(E)** Density plot of hucMSC-Exosomes. **(F)** Western blot of hucMSC-Exosome markers **(G)** Representative images of wound closure process of hucMSC-Exosomes treatment and PBS control. The scale bar is 4 mm. **(H)** Wound closure rate of hucMSC-Exosomes treatment and PBS control group. Two-tailed unpaired t-test (n=6). Error bar: mean standard deviation. Ns, not significant, *p < 0.05, ***p < 0.001, ****p < 0.0001. **(I)** H & E and Masson staining of wounds seven days after injury. Scale bar = 1mm. **(J)** Uptake of hucMSC-Exosome by skin wound *in vivo*. Scale bar, 500μm.

“HucMSC-Exosomes accelerate wound healing. (A) Schematic overview of the study design. (B) Microscope image of huc-MSCs. The scale bar is 500 µm. (C) Flow cytometry analysis of HLA, CD29, CD34, CD45, CD73, CD90 and CD105 expression on the huc-MSC surface. (D) Transmission electron microscopy image of hucMSC-Exosomes. Scale bar, 100 nm. (E) Density plot of hucMSC-Exosomes. (F) Western blot of hucMSC-Exosome markers (G) Representative images of wound closure process of hucMSC-Exosomes treatment and PBS control. The scale bar is 4 mm. (H) Wound closure rate of hucMSC-Exosomes treatment and PBS control group. Two-tailed unpaired t-test (n=6). Error bar: mean standard deviation. ns, not significant, *p < 0.05, ***p < 0.001, ****p < 0.0001. (I) H & E and Masson staining of wounds seven days after injury. Scale bar = 1mm. (J) Uptake of hucMSC-Exosome by skin wound *in vivo*. Scale bar, 500μm.”

The authors apologize for this error and state that this does not change the scientific conclusions of the article in any way. The original article has been updated.

