# Supplementary material for: Human umbilical cord mesenchymal stem cell-derived exosomes promote murine skin wound healing by neutrophil and macrophage modulations revealed by single-cell RNA sequencing
Source: Front Immunol. 2023 Mar 6;14:1142088. doi: 10.3389/fimmu.2023.1142088 (PMC10044346; doi:10.3389/fimmu.2023.1142088)
Supplement: Supplementary file 1 [file DataSheet_1.pdf]

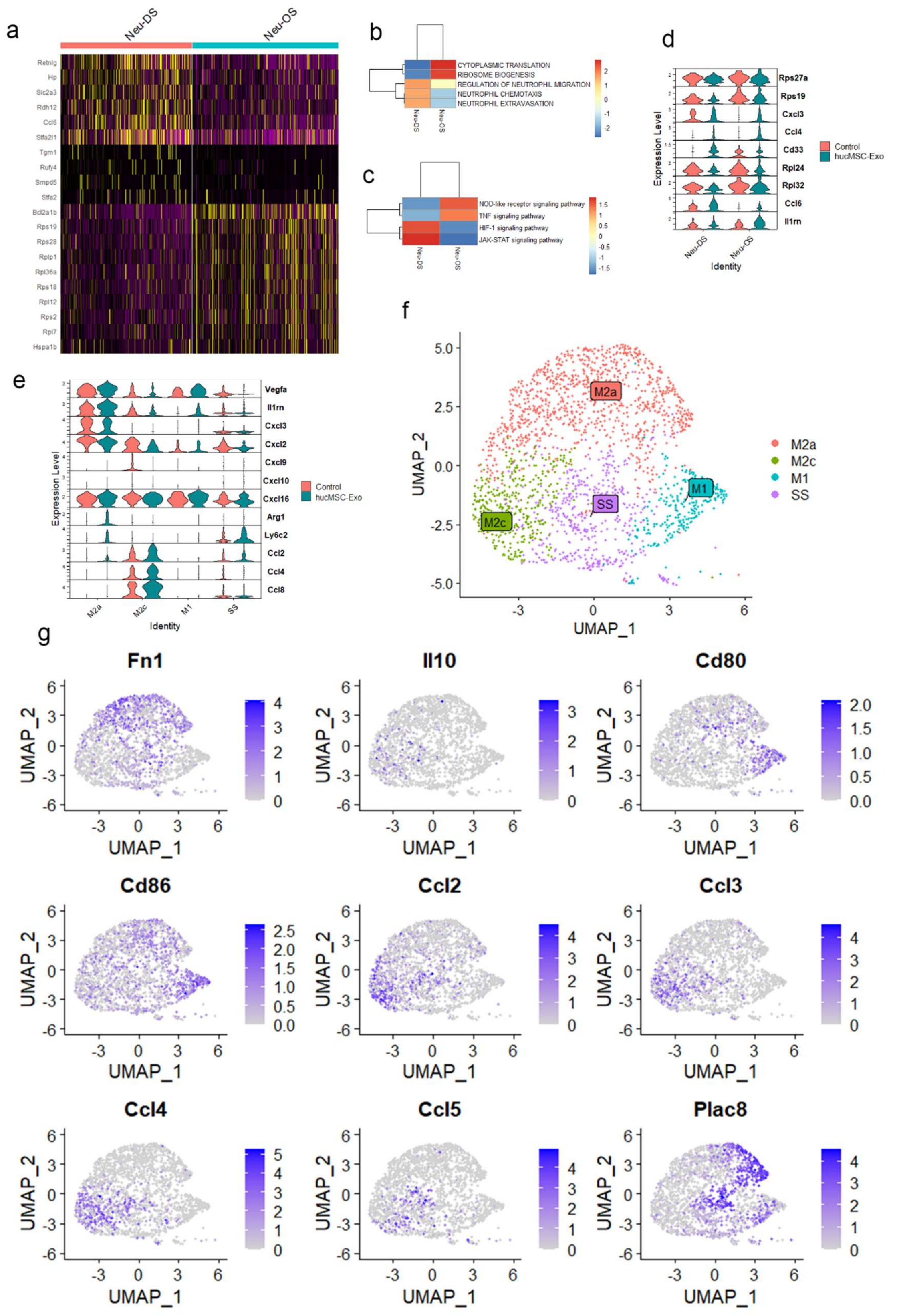

Supplementary Figure 1

a) Heatmap of top expressed genes in neutrophil subtypes
b) Heatmap of Gene Set Enrichment Analysis (GSEA) of genes expressed by neutrophils
c) Heatmap of Kyoto Encyclopedia of Genes and Genomes (KEGG) analysis of genes

expressed by neutrophils

d) Stacked violin plot of relevant differentially expressed genes (DEGs) of neutrophils following hucMSC-Exosomes exposure
e) Stacked violin plot of relevant differentially expressed genes (DEGs) of macrophages following hucMSC-Exosomes exposure
f) Combined 2D scatterplot based on UMAP reduction of macrophage coordinates
g) Scatterplot based on UMAP reduction of macrophages; cells are colored based on gene expression.

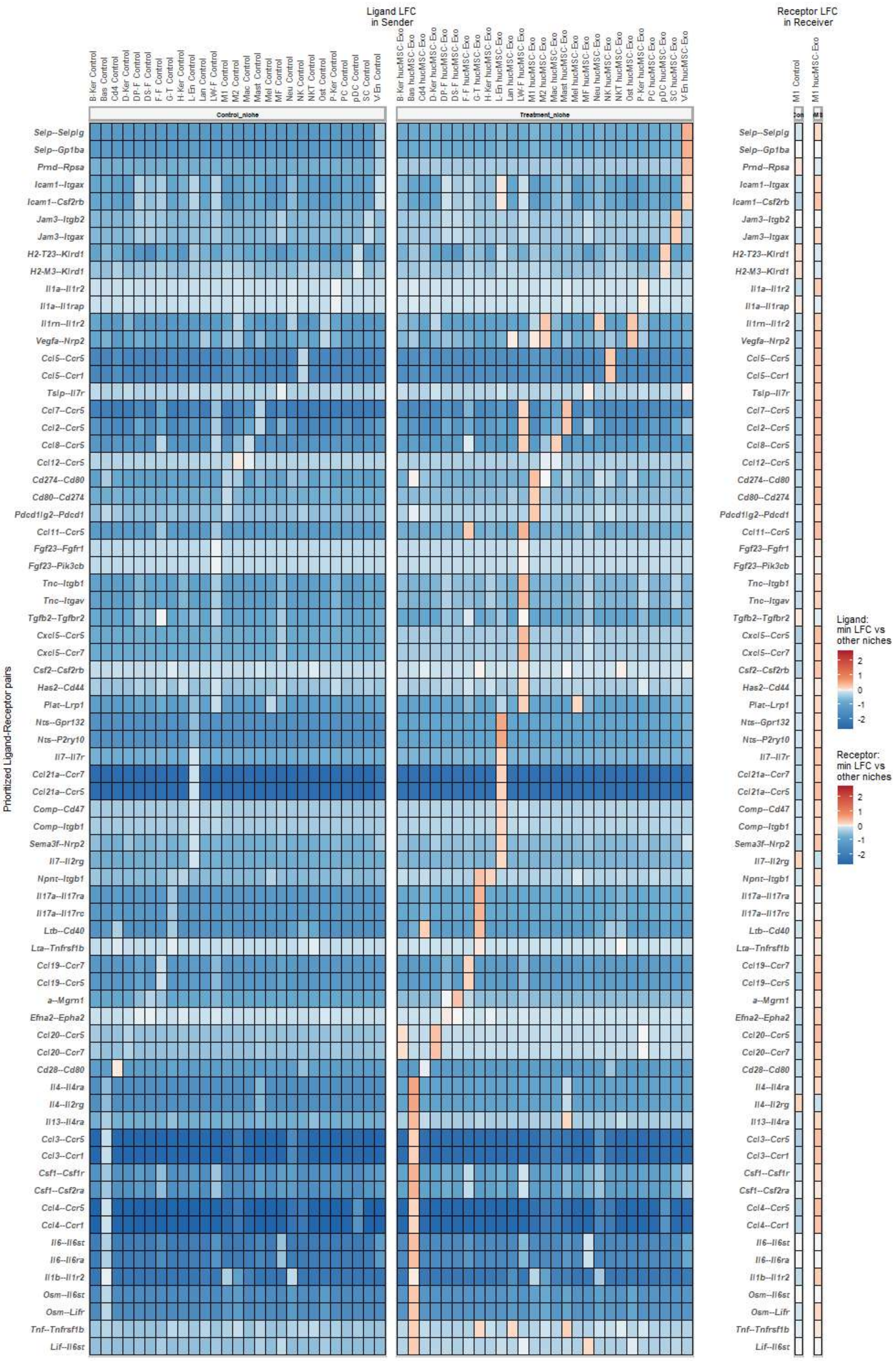

Supplementary Figure 2

Heatmap illustrating ligand-receptor expression comparison between the control group and the hucMSC-Exosomes treatment group for M1 macrophages (receiver).

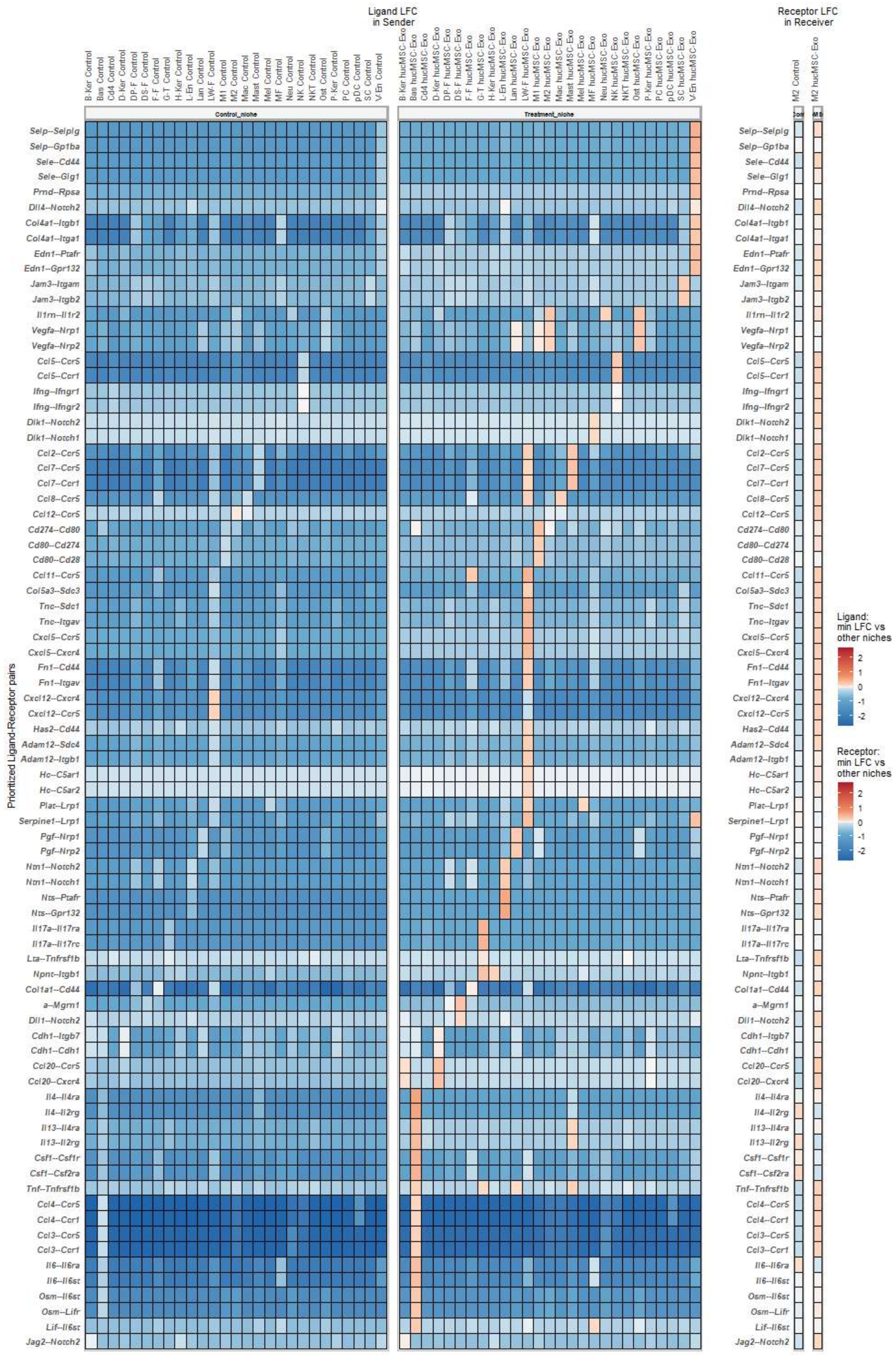

Supplementary Figure 3

Heatmap illustrating ligand-receptor expression comparison between the control group and the hucMSC-Exosomes treatment group for M2 macrophages (receiver).
